# Effects of Cadmium on Bioaccumulation, Bioabsorption, and Photosynthesis in *Sarcodia suiae*

**DOI:** 10.3390/ijerph17041294

**Published:** 2020-02-18

**Authors:** Tai-Wei Han, Chung-Chih Tseng, Minggang Cai, Kai Chen, Sha-Yen Cheng, Jun Wang

**Affiliations:** 1 Department of Environmental Biology and Fisheries Science, National Taiwan Ocean University, Keelung 20224, Taiwan; htw0620@scivision.com.tw; 2 Department of Dentistry, Zuoying Branch of Kaohsiung Armed Forces General Hospital, Kaohsiung 81357, Taiwan; caviton@gmail.com; 3 Institute of Medical Science and Technology, National Sun Yat-sen University, Kaohsiung 80424, Taiwan; 4 College of Ocean and Earth Science, Xiamen University, Xiamen 361102, China; mgcai@xmu.edu.cn; 5 Department of Biological Technology, Xiamen Ocean Vocational College, Xiamen 361102, China; wangjun@xmoc.edu.cn

**Keywords:** cadmium, bioaccumulation, bioabsorption, *Sarcodia suiae*, photosynthesis, respiration, photosynthetic pigments

## Abstract

This study investigated the changes in bioaccumulation, bioabsorption, photosynthesis rate, respiration rate, and photosynthetic pigments (phycoerythrin, phycocyanin, and allophycocyanin) of *Sarcodia suiae* following cadmium exposure within 24 h. The bioabsorption was significantly higher than the bioaccumulation at all cadmium levels (*p* < 0.05). The ratios of bioabsorption/bioaccumulation in light and dark bottles were 2.17 and 1.74, respectively, when *S. suiae* was exposed to 5 Cd^2+^ mg/L. The chlorophyll a (Chl-a) concentration, oxygen evolution rate (photosynthetic efficiency), and oxygen consumption rate (respiratory efficiency) decreased with increasing bioaccumulation and ambient cadmium levels. The levels of bioaccumulation and bioabsorption in light environments were significantly higher than those in dark environments (*p* < 0.05). In addition, the ratios of phycoerythrin (PE)/Chl-a, phycocyanin (PC)/Chl-a, and allophycocyanin (APC)/Chl-a were also higher in light bottles compared to dark bottles at all ambient cadmium levels. These results indicated that the photosynthesis of seaweed will increase bioaccumulation and bioabsorption in a cadmium environment.

## 1. Introduction

Red seaweed *Sarcodia suiae* grows in the intertidal or subtidal zone, and is widely distributed throughout Indo-western Pacific regions, including Taiwan and Japan. The *Sarcodia* family also includes species such as *S. montagneana* and *S. ceylanica* [1,2]. The large red algae *S. suiae* is available in all seasons and widely commercially cultured in Taiwan. *S. suiae* has a multiaxial growth pattern, and thallus blades composed of an outer cortex of anticlinal filaments [3,4]. Recently, *S. suiae* has been consumed as a health food because of its high concentration of micronutrients.

Human activity has significantly affected the distribution of heavy metals in the environment, including air, water, and sediment. Some heavy metals are essential elements for life, such as copper, zinc, and nickel, since they are important catalysts for enzyme metabolism [5]. In contrast, some heavy metals are not only nonessential elements for life but are also toxic such as mercury, cadmium, and lead. In general, nonessential and toxic heavy metals are not broken down by aquatic microorganisms and can migrate and accumulate among organisms, causing serious ecological problems [6]. Seaweed in coastal areas can become toxic due to exposure to a certain level of heavy metal contamination [7]. Seaweed’s strong bioaccumulation and bioabsorption of heavy metals makes it suitable to act as a biomarker of heavy metal pollution [8,9]. Generally, bioabsorption of heavy metal occurs on seaweed’s surface is the first stage, and then heavy metals pass through the cell and accumulate in the second stage [10].

Seaweed is known as one of the most important primary producers and the basis of the food chain in most aquatic ecosystems around the world [11,12]. Through photosynthesis, seaweed can use sunlight to convert water and carbon dioxide into sugar that feeds other organisms [13]. Some studies have reported that photosynthesis of seaweed is affected by heavy metals [14,15,16], since most seaweed habitats have been disturbed by intensive human activity [17,18]. Additionally, heavy metals that also change photosynthetic pigments include chlorophyll a (Chl-a), phycoerythrin (PE), phycocyanin (PC), and allophycocyanin (APC) [19,20,21]. The levels of these pigments are related to algal photosynthesis and environmental adaptation [22,23,24]. For example, magnesium, which is the central atom of chlorophyll a, can be replaced by heavy metals (e.g., mercury, copper, cadmium, nickel, zinc, and lead), leading to a breakdown in photosynthesis of algae and plant [25]. An evaluation experiment showed that PC and APC increased with light intensity while PE decreased in the body of *Nostoc sphaeroides* [26].

Cadmium is one of the most toxic heavy metals. Cadmium pollution has become a global issue for the ecosystem and environmental sustainability [27,28,29]. Cadmium displays a nutrient-like distribution in the oceans that closely follows the vertical profile of PO_4_^3−^, with depleted levels at the surface of the ocean and higher concentrations in intermediate and deep waters [29,30]. Like the other heavy metals, cadmium in the aquatic environment can be easily uptaken by phytoplankton [29,31]. Higher organisms can uptake cadmium in food and also directly absorb it from the environment. In order to maintain ecological security, it is necessary to understand the accumulation of cadmium in seaweed and the corresponding physiological reaction.

Although some studies reported heavy metal toxicity and bioaccumulation in seaweed, bioabsorption and the relationship between bioabsorption and bioaccumulation of heavy metals in seaweed are rarely discussed. Therefore, the aim of this study was to investigate the situation that the large red seaweed (*S. suiae*) exposes to different cadmium levels (control, 1 and 5 mg/L) with different exposure times (0, 1, 2, 3, 6, 12, 18, and 24 h), to measure its capacities of bioaccumulation and bioabsorption of cadmium. We also detected differences in photosynthesis rate; respiratory rate; and levels of Chl-a, PE, PC, and APC, to understand how these parameters change with toxic cadmium levels. These parameters can potentially be used as biomonitors to test for cadmium contamination in coastal water.

## 2. Materials and Methods

Seawater was pumped from the Keelung coast adjacent to the National Taiwan Ocean University and filtered through a gravel-and-sand bed by air-lifting. The water was aerated for 3 days before use. The cadmium test solution was prepared by dissolving Cd(NO_3_)_2_ (Merck, Darmstadt, Germany) in 1 L of distilled water to make a 1000 mg/L stock solution, which was then diluted to 1 and 5 mg/L, respectively. The cadmium concentrations of the test solutions were measured on a flame atomic absorption spectrometer (SpectrAA 240FS, Varian, Palo Alto, CA, USA). The experimental condition and the accuracy and precision of the analytical method were illustrated by Licate et al. (2004) [32].

Red seaweed (*Sarcodia suiae*) (4.585 ± 0.071 g) was obtained from Pingdong County, Taiwan. Seaweed was acclimated in fiberglass-reinforced plastic tanks at a salinity of 34‰ for two weeks before experimentation. The water temperature was maintained at 20 ± 1 °C, and dissolved oxygen (DO) was maintained at 7.83 ± 0.83 mg/L and pH values at 8.32 ± 0.04. During the acclimating period, ammonium nitrate (NH_4_NO_3_, 160 mg/L) and water-soluble phosphorus, P_2_O_5_, 4000 mg/L) were added to the seawater for nutrients. The mean (±SD) wet body weight did not significantly differ among treatments (*p* > 0.05).

The Cd^2+^ concentration was adjusted to 0 (control), 1, and 5 mg/L with the prepared stock solution at salinities of 34‰ seawater. Seaweed samples were placed into Pyrex glass BOD bottles (950 ± 0.8 mL) filled with a test Cd^2+^ solution. One piece of seaweed was placed in each bottle, which was then capped and placed in a water bath (20 ± 0.6 °C). There were five replicates for each salinity. The experiment lasted for 24 h, with a renewal of each test solution after 1, 2, 3, 6, 12, and 18 h. The same cadmium concentration bottles were divided into light (55 µmol protons/m^2^/s) and dark bottles. DO was measured when the test solution was renewed and at the end of the test. DO was measured with a YSI Model 5800 DO meter (Yellow Spring Instrument, Yellow Springs, OH, USA) together with a stirrer following air calibration with salinity compensation. When the test solution was renewed, water was sampled to measure the Cd^2+^ concentration. To determine whether there was bacterial growth on the inner walls of the bottle, three bottles of each test solution only (with no seaweed added) were monitored for DO. No significant differences in DO were found in any of the blanks during the sampling time (*p* > 0.05).

The measure condition of the photosynthesis and respiration rates was modified from the previous study by Fukumoto et al. (2019) [13]. The difference of the DO level and exposure time between the light and dark bottles was used to calculate the oxygen evolution rate (μmol O_2_/min/g algal wet weight) and oxygen consumption rate (μmol O_2_/min/g algal wet weight). 

Next, seaweed samples were washed with HCl 0.1 mol/L for 30 s, and an acid solution was used to measure the cadmium that the algal surface had absorbed. Seaweeds were then collected to detect the cadmium in the algal body. Acid washed seaweed samples were removed immediately and frozen at −20 °C. Next, samples were dried with a freeze-dryer (FD-20A2D, H.C.S., Taipei, Taiwan) at −60 °C. After 3 days, concentrated nitric acid (70%) was added to the samples and digested in a microwave-accelerated reactor (MARS Xpress, CEM, Matthews, NC, USA). The seaweed Cd^2+^ ions were determined using a flame atomic absorption spectrophotometer (SpeactAA 240FS, VARIAN, Palo Alto, Santa Clara, CA, USA). 

Dried seaweeds samples were weighted, and PBS (phosphate-buffered saline) was added. The samples were homogenized twice at 13 °C with a model FastPrep-24 5G (MP Biomedicals, Santa Ana, CA, USA) and centrifuged at 3000× *g* for 5 min at 4 °C with a Model 5403 centrifuge (Thermo Fish Scientific, Waltham, MA, USA). We detected the absorbance of the supernatant at wavelengths of 562, 615, and 652 nm using an ELISA reader (VersaMax Microplate Reader, Molecular Devices, Silicon Valley, San Francisco, CA, USA). We calculated the PE, PC, and APC contents with the algal wet weight using the following formula:

(1) $$\mathrm{PE}\;(\mathrm{mg}/g)\;=\;\frac{{Abs}_{562}\;-\;\left(2.41\;\times \;\mathrm{PC}\right)\;-\;(0.849\;\times \;\mathrm{APC})}{9.62}$$

(2) $$\mathrm{PC}\;(\mathrm{mg}/g)\;=\;\frac{\;{Abs}_{615\;}-\;({0.474\;\times \;Abs}_{652})}{5.34}$$

(3) $$\mathrm{APC}\;(\mathrm{mg}/g)\;=\;\frac{{Abs}_{652}\;-\;({0.208\;\times \;Abs}_{615})}{5.09}$$

Dried seaweeds samples were weighted, and distilled water was added. The samples were homogenized twice at 13 °C with a model FastPrep-24 5G (MP Biomedicals, Santa Ana, CA, USA) and centrifuged at 3000× *g* for 5 min at 4 °C with a Model 5403 centrifuge (Thermo Fisher Scientific, Waltham, MA, USA). The supernatant was removed and 90% acetone was added, incubating 2 h at 4 °C. We detected the absorbance at 630, 647, 664, and 750 nm, with a spectrophotometer (GE Healthcare Life Science, Buckinghamshire, UK). The following formula was used to calculate the concentration of Chl-a using acetone volume and algal wet weight [33].

(4) $$\mathrm{Chl}-a\;(\text{µ}g/g)\;=\;{11.85\;(Abs}_{664}{-\;Abs}_{750})\;-\;{1.54\;(Abs}_{647}\;{-\;Abs}_{750})\;-\;{0.08\;(Abs}_{630}\;{-\;Abs}_{750})$$

All data were assessed by one- and two-way ANOVA. If the significant difference was indicated at the 0.05 level, then Duncan’s multiple-range test was used to identify significant differences among treatments. Statistical significance of all tests was accepted at the *p* < 0.05 level.

## 3. Results

**Figure 1:**
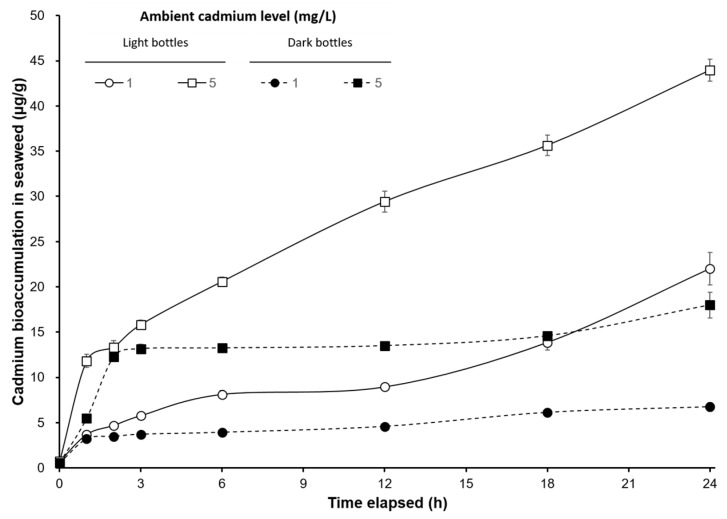
Time-course changes of cadmium bioaccumulation (µg/g) on *S. suiae* under different cadmium concentrations (1 and 5 mg/L) exposure in light and dark bottles.

The cadmium bioaccumulation level in the large red algal *S. suiae* (control) was 0.034 ± 0.007 μg/g, while the seaweeds in light bottles exposed to 1 Cd^2+^ mg/L solution for 1, 2, 3, 6, 12, 18, and 24 h, the cadmium levels in the algal body were 3.695, 4.691, 5.768, 8.116, 8.938, 13.847, and 21.997 μg/g, respectively. The cadmium levels in algal body, respectively, were 11.833, 13.335, 15.817, 20.579, 29.411, 35.636, and 43.938 μg/g, during the same period time, when *S. suiae* exposed to 5 Cd^2+^ mg/L solution. The cadmium bioaccumulation of *S. suiae* showed a significant positive correlation with exposure time in light bottle (*p* < 0.05). For the seaweeds in dark bottles exposed to 1 Cd^2+^ mg/L for 1, 2, 3, 6, 12, 18, and 24 h, the cadmium levels in the algal body were 3.206, 3.471, 3.716, 3.933, 4.583, 6.128, and 6.745 μg/g, respectively. The cadmium levels in the algal body were 5.486, 12.266, 13.156, 13.246, 13.497, 14.586, and 17.984 μg/g, after exposure to a 5 Cd^2+^ mg/L solution with the same exposure time. The cadmium levels after exposure to 5 mg/L were significantly higher than those after exposure to 1 mg/L during each period and in the light and dark bottles (*p* < 0.05). The bioaccumulation of cadmium in *S. suiae* increased with exposure time and each ambient cadmium concentration in both light and dark bottles (Figure 1).

**Figure 2:**
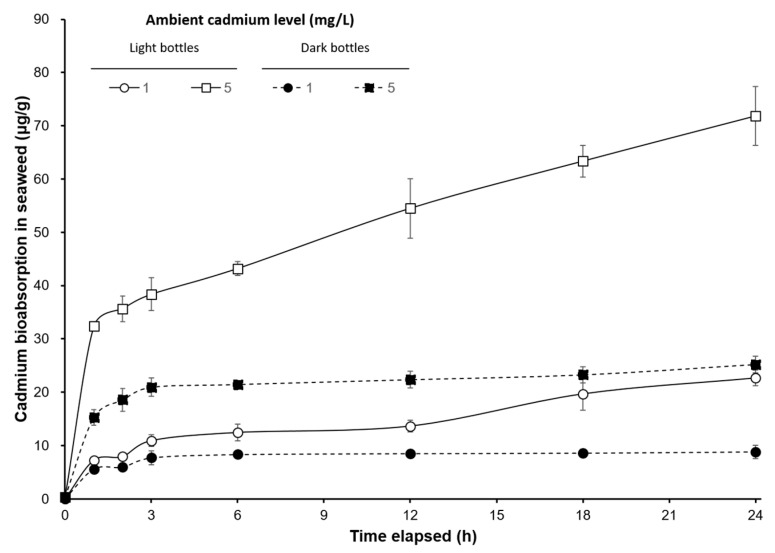
Time-course changes of cadmium bioabsorption (µg/g) on *S. suiae* under different cadmium concentrations (1 and 5 mg/L) exposure in light and dark bottles.

The cadmium bioabsorption levels of the seaweed in dark bottles were significantly lower than those in light bottles for each ambient cadmium level (*p* < 0.05). The bioabsorption levels of *S. suiae* exposed to 1 Cd^2+^ mg/L in light bottles were 7.253, 7.900, 10.925, 12.457, 13.659, 19.682, and 22.667 μg/g, after exposure times of 1, 2, 3, 6, 12, 18, and 24 h, respectively. At the same exposure time with 5 Cd^2+^ mg/L, the surface cadmium levels were 32.350, 35.667, 38.367, 43.200, 54.500, 63.350, and 71.825 μg/g, respectively. There were significant differences within the exposure time (*p* < 0.05). The surface cadmium levels of *S. suiae* in dark bottles were 5.625, 5.974, 7.728, 8.351, 8.525, 8.626, and 8.859 at 1 Cd^2+^ mg/L, respectively, and they were 15.275, 18.575, 20.950, 21.475, 22.375, 23.275 and 25.225 at 5 Cd^2+^ mg/L, respectively (Figure 2). The ratio of bioabsorption/bioaccumulation in the light and the dark bottles was 1.58 and 1.75, respectively, when exposed at 1 Cd^2+^ mg/L. The ratios of bioabsorption/bioaccumulation in the light and the dark bottles were 2.17 and 1.74, respectively, when exposed to 5 Cd^2+^ mg/L solution.

**Figure 3:**
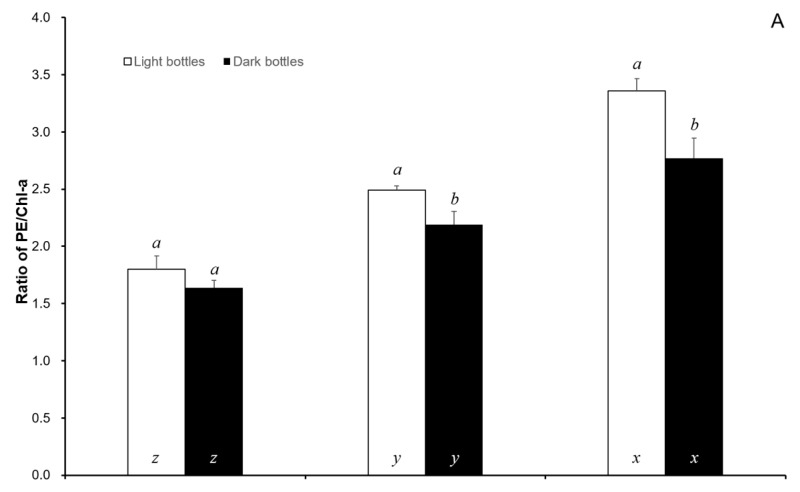

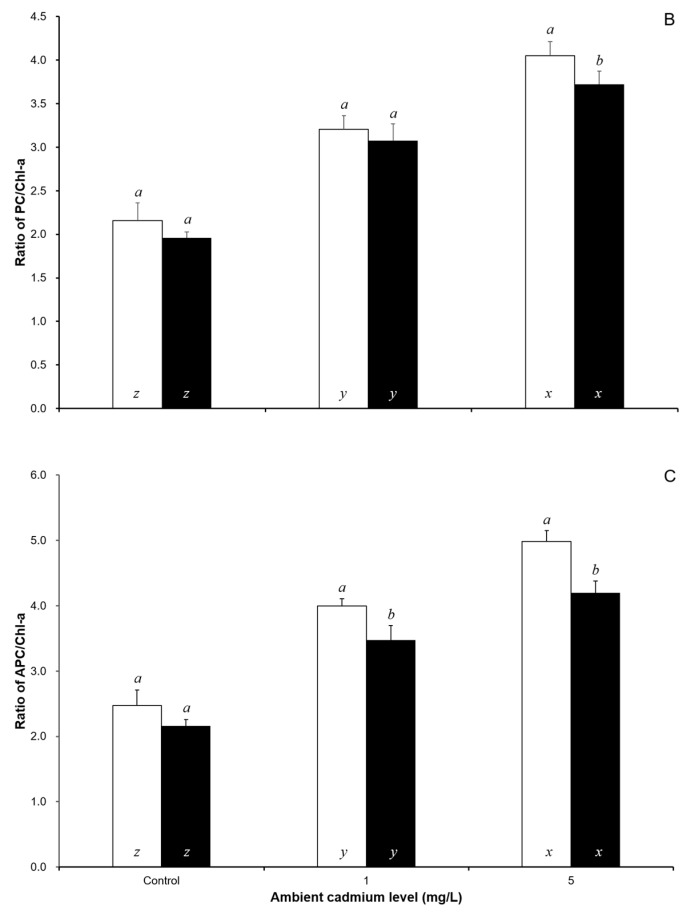
The ratios of PE/Chl-a (**A**), PC/Chl-a (**B**), and APC/Chl-a (**C**), in *S.suiae,* following 24 h exposure with control, 1, and 5 Cd^2+^ mg/L. Data in the same ambient cadmium level having different letters (*a* and *b*) are significantly different (*p* < 0.05) between light and dark bottles, and data in the same light or dark bottles having different letters (*x*, *y* and *z*) are significantly different (*p* < 0.05) among different ambient cadmium levels; Chl-a—chlorophyll a; PE—phycoerythrin; PC—phycocyanin; APC—allophycocyanin.

The Chl-a contents in the algal body decreased with ambient cadmium levels. There was no significant difference between the different ambient cadmium levels. The PE, PC, and APC contents significantly increased with the ambient cadmium levels (*p* < 0.05). For the seaweeds exposed to cadmium after 24 h in light bottles, the ratios of PE/Chl-a, PC/Chl-a, and APC/Chl-a were 1.800, 2.155, and 2.475 in the control, respectively; 2.493, 3.208, and 3.998 in the 1 Cd^2+^ mg/L treated samples, respectively; and 3.357, 4.050, and 4.986 in the 5 Cd^2+^ mg/L treated samples, respectively. The ratios of PE/Chl-a, PC/Chl-a, and APC/Chl-a were not significantly different from those of the control (*p* > 0.05). However, there were significant differences within the 5 Cd^2+^ mg/L-treated samples (*p* < 0.05) (Figure 3). The ratios of PE/Chl-a, PC/Chl-a, and APC/Chl-a also showed significant differences between control and cadmium exposure (*p* < 0.05).

**Figure 4:**
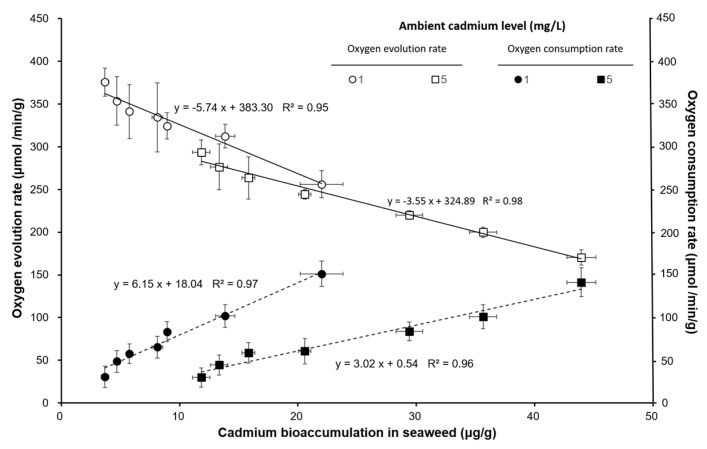
The relationship between the oxygen evolution rate (μmol/min/g) and oxygen consumption rate (μmol/min/g) with cadmium content on seaweed bioaccumulation (μg/g) in *S. suiae* exposed to 1 and 5 Cd^2+^ mg/L.

**Figure 5:**
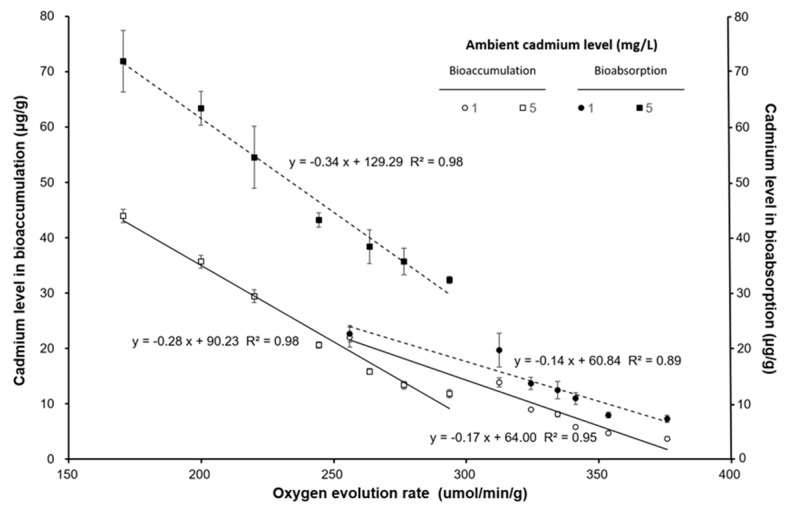
The relationship between the cadmium content in seaweed bioaccumulation (μg/g) and bioabsorption (μg/g) with the oxygen evolution rate (μmol/min/g) on *S. suiae* exposed to 1 and 5 Cd^2+^ mg/L.

**Figure 6:**
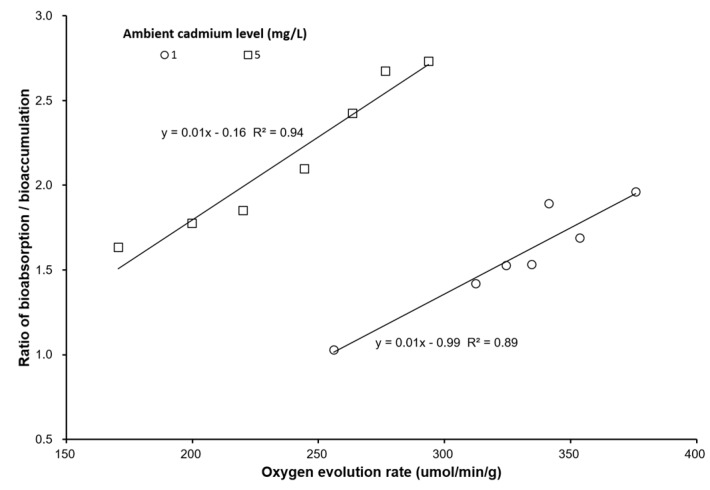
The relationship between the ratio of bioabsorption/bioaccumulation and the oxygen evolution rate (μmol/min/g) on *S. suiae* exposed to 1 and 5 Cd^2+^ mg/L.

The oxygen evolution rate (μmol/min/g) and oxygen consumption rate (μmol/min/g) decreased with increasing ambient cadmium concentration and exposure time. The oxygen evolution rate was significantly lower than that of the control and the 1 Cd^2+^ mg/L group (*p* < 0.05). The oxygen consumption rate was not significantly different among the varied exposure times (*p* > 0.05). Figure 4 shows that the oxygen evolution rate and oxygen consumption rate had an approximates linear relationship with the cadmium level in seaweed bioaccumulation. The oxygen evolution rate decreased when the cadmium bioaccumulation in seaweed was increased, but the oxygen consumption rate increased when the cadmium bioaccumulation in seaweed was increased. The cadmium bioaccumulation and bioabsorption had a negative relationship with the oxygen evolution rate (Figure 5). The ratio of bioabsorption/bioaccumulation was 1.03–1.96 and 1.63–2.73 for seaweed exposed to 1 and 5 mg/L cadmium environment, respectively. The ratio of bioabsorption/bioaccumulation increased with the oxygen evolution rate (Figure 6).

## 4. Discussion

Some heavy metals are essential for organisms, but cadmium is not an essential element for most organisms and is often toxic. Cadmium is one of the most common and widespread heavy metals found in the environment and adversely affects seaweed and phytoplankton [34,35,36,37,38].

Cadmium can affect the growth, cell size, and Chl-a content of the freshwater green algae *Chlorolobion braunii*, with the EC_50_ value of 1.6 µM [39]. Seaweeds absorb heavy metals from the environment, and this process is affected by various mechanisms that take place in microscale [40]. Heavy metals are absorbed that occur on the seaweed surface, which then pass through the seaweed cell and accumulate in the algal body [10]. Proteins and glycoproteins in the algal cell wall are the first barriers to combining heavy metals. The seaweed surface presents major functional groups, including hydroxyl, carboxylate, sulfate, and phosphate groups, which can absorb heavy metals. Moreover, the algae cell wall is rich in alginic acid, which has a high absorption capacity of heavy metals [41,42]. These compounds are very important in bioaccumulation and bioabsorption [43,44].

The cell wall of algae provides protection from metal ions [45]. Our result indicated that the bioabsorption was significantly higher than the bioaccumulation at the same ambient cadmium levels. Both the bioaccumulation and bioabsorption in *S. suiae* increased with increasing ambient cadmium levels. However, the bioaccumulation and bioabsorption in the light bottles were significantly higher than those in the dark bottles. The result indicated that the photosynthesis will increase cadmium absorption on seaweed surface and accumulation on seaweed body. The oxygen evolution rate and oxygen consumption rate indicate photosynthesis efficiency and respiration efficiency, respectively. This study demonstrated that bioaccumulation and bioabsorption of cadmium were also related to the oxygen evolution rate. Xia et al. (2004) found the photosynthetic O_2_ evolution was inhibited by ambient cadmium and copper in red seaweed *Gracilaria lemaneiformis* [14]. Our results also showed that the oxygen evolution rate had an increasing ambient cadmium level. On the contrary, the respiration rate of *S. suiae* increased when the ambient cadmium level increased.

Figure 2 shows bioaccumulation and bioabsorption of *S. suise* in dark bottles quickly increased within 3 h, but was constant after 3 h. However, bioaccumulation and bioabsorption in seaweed in the light bottles continued to increase after 3 h. The ratio of bioabsorption/bioaccumulation also increased with an increasing oxygen evolution rate. This result shows that photosynthesis efficiency influences the bioabsorption and bioaccumulation of cadmium in seaweed.

Bastosa et al. (2019) also found similar results in *Ulva ohnoi* [46]. This result may be caused by Cd ion affecting Ca ion exchange and Mn ion cofactors that affect the Hill reaction and inhibit the oxygen evolution rate [47,48]. Cd also has several effects, such as interference in the electron transfer between plastoquinones [48,49]. Not only is the photosynthesis efficiency affected, but respiratory efficiency is also affected by cadmium. Lu et al. (2018) indicated that the oxygen evolution rate and oxygen consumption rate decreased with increasing cadmium in brown algae *Sargassum thunbergii* [50]. Similar results were also observed in our study. Moreover, we also found that bioabsorption was significantly higher than bioaccumulation at the same ambient cadmium concentration and in either light or dark environments. Thus, bioabsorption and bioaccumulation increase with increasing photosynthesis efficiency.

Photosynthetic pigments capture light energy, which is then passed on to chlorophylls during photosynthesis, and include PE, PC, and APC, which are red, blue, and light-blue pigments, respectively [51,52,53,54]. When exposed to cadmium, the agarophyte *Gracilaria domingensis* had decreased levels of Chl-a and phycobiliproteins after 4 days of exposure [19]. Environmental light changes the photosynthetic pigments. When cells are grown in continuous light, there is a 25% reduction in total pigment in the light-/dark-grown culture and a 50% reduction in the dark-grown culture [22]. We also showed that Chl-a decreased but photobiliproteins increased with increasing levels of ambient cadmium. This result may be caused by exposure time and cadmium levels. In our study, the exposure time was 24 h at a cadmium concentration of 5 mg/L, which is significantly lower than that in the other studies. However, the ratios of PE/Chl-a, PC/Chl-a, and APC/Chl-a increased with increasing ambient cadmium levels. These above ratios showed significant differences between control and cadmium exposure. In 5 Cd^2+^ mg/L exposure, the ratios PE/Chl-a, PC/Chl-a, and APC/Chl-a, in the light bottles, were significantly higher than those in the dark bottles. This result may be caused by the inhibition of photosynthesis; the algal photosynthetic pigments increased to recover the loss caused by cadmium exposure.

## 5. Conclusions

This study showed the bioabsorption and bioaccumulation of cadmium were related to seaweed’s photosynthesis. In light bottles, we found higher bioabsorption and bioaccumulation of cadmium than dark bottles. These results strongly showed that cadmium affects photosynthesis efficiency, respiratory efficiency, and photosynthetic pigments of *S. suiae*. The high bioabsorption of *S. suiae* can eliminate environmental cadmium quickly. The ambient cadmium will change the ratio of PC/Chl-a, PC/Chl-a, and APC/Chl-a in *S. suiae*. These ratios in seaweed can be used as biomonitors for short-term cadmium exposure.
